# Immune-related RELT drives clear cell renal cell carcinoma progression through JAK/STAT signaling pathway activation

**DOI:** 10.3389/fimmu.2025.1659119

**Published:** 2025-11-25

**Authors:** Yini Wang, Yingying Yang, Tianqi Wang, Xiaohong Ma, Jitao Wu

**Affiliations:** 1 Yantai Affiliated Hospital of Binzhou Medical University, Yantai, Shandong, China; 2 Department of Urology, The Affiliated Yantai Yuhuangding Hospital of Qingdao University, Yantai, Shandong, China

**Keywords:** ccRCC, RELT, tumor immune microenvironment, JAK/STAT pathway, T cell immunity

## Abstract

**Objective:**

The experiment aims to verify the function of Tumor Necrosis Factor Receptor Superfamily Member 19L (RELT) in clear cell renal cell carcinoma (ccRCC).

**Methods:**

The relationship between differential expression of RELT in ccRCC and clinical prognosis was investigated based on data from the Gene Expression Omnibus database (GEO) and The Cancer Genome Atlas (TCGA) databases. Ex vivo and *in vivo* experiments were applied to validate the function of RELT in ccRCC. The pathways through which RELT exerts its function were explored using analyses such as Gene Ontology (GO), Kyoto Encyclopedia of Genes and Genomes Enrichment Analysis (KEGG), and Gene Set Enrichment Analysis (GSEA). In addition, we applied the algorithms xCELL, Estimating the Proportion of Immune and Cancer cells (EPIC), CIBERSORT, Tumor Immune Estimation Resource (TIMER), and Tumor Immune Dysfunction and Exclusion (TIDE) to analyze the effect of RELT on the ccRCC tumor immune microenvironment.

**Results:**

RELT is highly expressed in ccRCC tissues and portends poor prognosis. Functional assays indicate that RELT promotes malignant biological behavior in ccRCC cells. Subsequently, enrichment analysis revealed that RELT functions mainly through humoral immunity, cellular chemotaxis, and cytokine regulation and may serve as a molecule for predicting prognosis in ccRCC. Immune infiltration analysis showed that RELT was significantly associated with immune cells such as B Cells, CD8+ T Cells, CD4+ T Cells, and Macrophages and may affect the tumor immune microenvironment of ccRCC by influencing macrophages.

**Conclusion:**

RELT promotes the development of ccRCC and may play a role in regulating the tumor immune microenvironment, which affects the prognosis of ccRCC patients, and RELT may become a new biomarker associated with immune infiltration in ccRCC.

## Graphical Abstract

Flowchart of the Article.

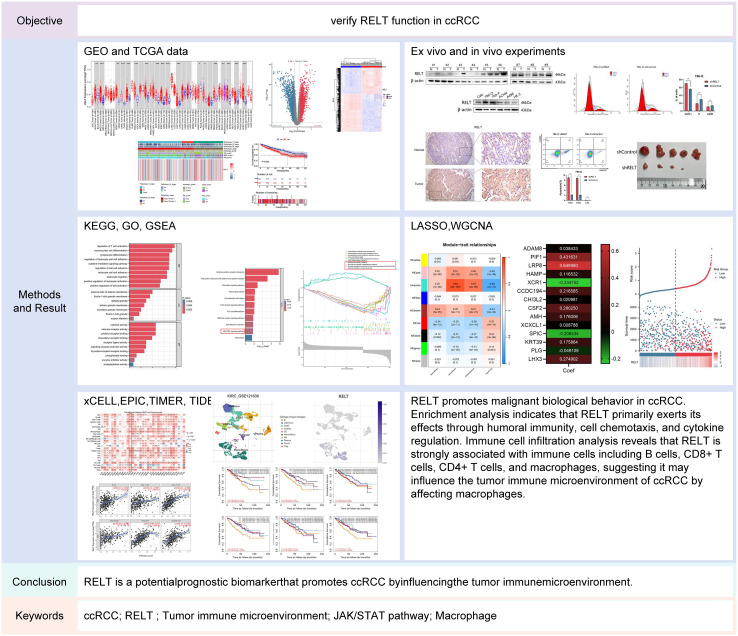

## 1 Introduction

The global population aged 65 and above is projected to reach 1.6 billion by 2050. The elderly constitute a high-risk group for urinary tract tumors such as bladder cancer, kidney cancer, and prostate cancer, resulting in an increasingly heavy medical burden (1). Renal cell carcinoma (RCC) is one of the most common urinary tract tumors, categorized into ccRCC, kidney chromophobe (KICH), and kidney renal papillary cell carcinoma (KIRP). ccRCC is the main subtype of RCC, accounting for 75-80% of all RCC cases (2). Its incidence is increasing every year, and it is usually diagnosed between the ages of 50 and 70 years (3). Major risk factors for the development of ccRCC include smoking, hypertension, obesity, and chronic kidney disease (3). Approximately 3-5% of these RCCs are associated with hereditary renal cancer syndromes such as Von Hippel-Lindau disease (4). Surgery remains the mainstay of treatment for ccRCC due to its resistance to radiotherapy and chemotherapy. However, approximately 30% of patients experience recurrence or metastasis after surgery (5). VEGF-targeted therapy is the first-line treatment option for patients with metastatic ccRCC, but many patients exhibit primary resistance or acquired drug resistance (6). Cancer-specific survival and overall survival (OS) are worse in ccRCC compared to KIHC and KIRP. ccRCC has a high level of immune cell infiltration (2), and thus ccRCC is the subtype of RCC that responds best to immunotherapy and targeted therapies (2). Immunotherapy is also known as an important way to treat ccRCC, and the search for new targets of immunotherapy for ccRCC has become an urgent problem to be solved.

RELT is a member of the tumor necrosis factor superfamily (TNFRSF), RELT is expressed in the lymphoid tissue protein family, and Receptor Expressed In Lymphoid Tissues Like 1 (RELL1) and Receptor Expressed In Lymphoid Tissues Like 2 (RELL2) are proteins homologous to RELT (7). RELT has been associated with myeloma bone lesions, enamel development of teeth, cardiovascular disease, blood pressure regulation, and myocardial infarction (7, 8). RELT is also closely associated with cancer, and available reports have shown that increased expression of RELT in esophageal squamous cell carcinoma (ESCC) correlates with a poor prognosis for the patients (9). RELT is able to activate the NF-κB pathway, which promotes cancer progression and is associated with activation of the p38 and JNK MAPK pathways (7, 9). RELT acts in PRAD (prostate cancer) by correlating with the expression of immune checkpoints and dysfunction of T cells, dendritic cells, and neutrophils (10). It has been reported that RELT acts as a negative regulator of the early stages of T-cell activation and may promote T-cell apoptosis by inhibiting the T-cell response, thereby enhancing immune escape from tumors (7, 11). Several studies have shown that aberrant expression of RELT is associated with immune escape from cancer, including ESCC and PRAD (9, 10). However, the expression, mechanism, and clinical significance of RELT in ccRCC remain unclear.

In this study, we evaluated the aberrant expression, clinicopathological features, and predictive ability of RELT for prognosis in a variety of cancers by databases such as TCGA, GEO, GEPIA (Gene Expression Profiling Interactive Analysis), and Kaplan-Meier map (K-M) mapping online platforms. Firstly, the ability of RELT to affect proliferation, migration, invasion, cell cycle, and apoptosis in ccRCC and to correlate with the JAK/STAT pathway was validated. Secondly, the collated and collected TCGA data were applied to RStudio for multiple enrichment analyses of RELT. In addition, we applied multiple online platforms, including TIMER, GEPIA, and Tumor-Immune System Interaction Database (TISIDB), to conduct in-depth exploration of RELT-related immune mechanisms, which were subsequently validated experimentally.

## 2 Materials and methods

### 2.1 The cancer genome atlas database

We applied the TCGA database (https://genome-cancer.ucsc.edu/) to obtain detailed information about ccRCC patients, including RNA-seq expression data, prognostic information, and relevant clinicopathologic data. Analysis of RELT expression and prognostic level analysis. The relationship between the RELT gene and the tumor immune microenvironment in renal clear cell carcinoma was explored by applying ESTIMATE, tumor purity, and ssGSEA analysis. Performance was also assessed qualitatively and quantitatively using the area under the receptor operating characteristic (ROC) curve (AUC).

### 2.2 Gene expression omnibus database

In this paper, we applied the GSE53757 transcriptomics dataset from the GEO database, created and maintained by the National Center for Biotechnology Information (NCBI) (https://www.ncbi.nlm.nih.gov/geo/), to explore the expression of RELT in ccRCC tissues and matched normal kidney tissue gene chips.

### 2.3 K-M plotter

We applied the K-M plotter online tool to 530 ccRCC samples to plot survival curves to assess the survival as well as the prognosis of RELT from various aspects.

### 2.4 Cell lines and ccRCC clinical samples

ccRCC lines (Caki, 786-O, 769-P, ACHN, A498, and HK-2) were purchased from the cell bank of the Chinese Academy of Sciences. RPMI 1640 culture medium (Thermo Fisher Scientific, USA) was applied, and both were supplemented with 10% fetal bovine serum (FBS), as well as 1% penicillin and streptomycin for culture. The cells were cultured using a constant-temperature incubator at 37°C with 5% CO_2_. In addition, we selected nine pairs of cancer tissues and their paracancerous tissues from patients with renal clear cell carcinoma at Yantai Yuhuangding Hospital to extract cells for Western blot experiments.

### 2.5 T cell isolation

Perform T cell isolation within one hour of fresh blood collection. Dilute blood 1:1 with PBS containing 2% FBS, then centrifuge for immune cell separation using Ficoll. After collecting the immune cell pellet, sort T cells using a cocktail antibody-coated magnetic bead system. Finally, culture T cells in 24-well plates supplemented with IL-2 and activators. Add IL-2 to activate T cells at each passage. Cells are cultured in a 37 °C, 5% CO_2_ incubator.

### 2.6 Western blot

Protein blot cells were lysed with RIPA lysis buffer. Protein lysates were separated by sodium dodecyl sulphate-polyacrylamide gel electrophoresis and transferred onto polyvinylidene difluoride (PVDF) membranes (Beyotime, China) with a pore size of 0.22 μm. The PVDF membrane was incubated overnight at 4 °C with the following primary antibody: anti-RELT (#DF8712, Affinity, China); anti-JAK2 (#AF6022, Affinity, China); anti-Phospho-JAK2 (#AF3024, Affinity, China); anti-STAT3 (#AF6294, Affinity, China); anti-Phospho-STAT3 (#AF3293, Affinity, China), anti-β-actin (#S0B0005, STARTER, CHINA). Wash 3 solution was applied with Tris buffered saline with 0.1% Tween^®^20 decontaminant (TBST buffer), and after washing every 10 min for a total of three times, the PVDF membrane was incubated with goat anti-mouse secondary antibody. The signals were then visualized using a Tanon-5200 ECL system (Tanon, China). The results were quantified and normalized using ImageJ.

### 2.7 Immunohistochemical analysis

We performed immunohistochemical experiments on the cancerous tissues and their paracancerous tissues of 59 pairs of patients with renal clear cell carcinoma from Yantai Yuhuangding Hospital. All paraffin specimens were sectioned at 0.4 mm intervals, and the sections were baked at 65°C for 1 hour and then dewaxed. After dewaxing, 10 mM pH 6.0 citrate buffer was applied to repair the antigen. After applying anti-RELT (#DF8712, Affinity, China) for overnight incubation, DAB reagent and hematoxylin (Solarbio, China) were applied for staining, and finally the sections were dehydrated and sealed. Staining was observed and recorded using a microscope. The Immunoreactivity Scoring System (IRS) was applied to carry it out.

### 2.8 Cell line construction for knockdown of RELT

Shanghai Genechem Co., Ltd. (China) directly packaged small hairpin RNA and disordered shRNA into lentiviral particles with puromycin resistance-shRELT as well as shNC for infection of 786-O and 769-P cells and performed WB experiments to determine RELT levels to validate the knockdown efficiency as well as to establish stable transducing strains.

### 2.9 Determination of cell proliferation

1×10^3^ cells/well were inoculated in 6-well and 96-well plates, respectively, and the 96-well plates were cultured overnight, and then cell counting kit-8 (CCK-8) (Beyotime, China) was applied to detect the proliferation. 6-well plates were cultured for 10 days and then stained with crystal violet (Beyotime, China). Cell proliferation was detected by all the above methods. Plate cloning assay to determine cell proliferation.

### 2.10 Cell cycle assay

Cells were harvested, fixed in anhydrous ethanol, and stored at 4°C overnight. Cells were washed once in PBA, followed by staining with PI and incubation in the dark for 30 min. Cell cycle analysis was assessed on a MoFlo XDP flow cytometry sorter (Beckman Coulter, CHINA), and collected cell cycle data were analyzed using ModFit LT.

### 2.11 Scratch assay to detect cell migration

After inoculation of cells (8×10^5^ cells/well) into 6-well plates for wall attachment, the culture medium was replaced with FBS-free 1640 medium for scratching, and photographs were taken of the scratching status of cells at the same location at 0h, 12h, 24h, 36h, and 48h. The scratch area was subsequently measured as well as normalized using ImageJ.

### 2.12 Transwell assay to detect cell migration and invasion

The chambers in which cell invasion was measured were lined with 60 μL of Matrigel diluted 1:9 with FBS-free 1640 medium and incubated at 37 °C for 1 h. This step was not required for experiments to detect migration. After adding 700 μL of 1640 medium containing FBS in a 24-well plate into a Transwell chamber, 200 μL of FBS-free cell suspension with appropriate cell numbers (786-O 2×10^4^, 769-P 4×10^4^) was added to the chamber. After 24 h of incubation, the cells at the bottom of the Transwell chamber were fixed with methanol for 15 min and stained with crystal violet for 3 hours. Finally, the number of migrating or invading cells was counted using ImageJ.

### 2.13 Apoptosis and co-culture apoptosis detection

Apoptosis was detected with the Annexin V-APC/7-AAD Apoptosis Kit (Thermo Fisher Scientific, USA). Collected cells and supernatant culture medium, after resuspension, were incubated with 5 μL of Annexin V-APC and 10 μL of 7-AAD for 15 min at 25 °C in the dark. Finally, apoptosis levels were assessed using a Moflo XDP flow cytometric sorter (Beckman Coulter, CHINA), and data were analyzed using FlowJo.

For apoptosis detection in tumor cell-T cell co-culture, first seed 2 × 10^5^ tumor cells in a 6-well plate and allow them to adhere overnight. The following day, seed T cells cultured for 4 days at a 1:2 ratio into the tumor cell wells and add IL-2 to activate the T cells. After 24 hours of co-culture, T cells were removed using CD3-Pacific Blue (#300329, BioLegend, CHINA), and tumor cell apoptosis was assessed using the AnnexinV-APC/7-AAD Apoptosis Kit.

### 2.14 Tumor formation experiment in nude mice

Four-week-old BALB/c male nude mice were purchased from Shandong Luye Pharmaceutical Co. Animal experiments were approved by the Ethics Committee of Yantai Yuhuangding Hospital, affiliated with Qingdao University. To investigate the effect of RELT on tumor cell tumorigenesis in nude mice subcutaneously. Mice were randomly divided into two groups: a control group (shNC) and a transfected shRELT group (shRELT), with 5 mice in each group, and a total of 1×10^7^ cells were injected subcutaneously. Tumor length and tumor width were recorded every 4 days, and tumor volume was calculated using the following formula: volume (mm^3^) = 1/2 length × width. Mice were executed after 21 days; tumors were removed and weighed. And perform IHC experiments on mouse tumors. Mouse tumors were immunohistochemically stained for Ki67 (#ab15580, Abcam, UK). All experimental animals were monitored every 2–3 days by designated personnel throughout the study period. During the 21-day rearing period, the mice exhibited no signs of distress, remained in good health, and showed no significant weight loss. Tumor volume was consistently maintained below 1.5 cm in diameter. Prior to euthanasia, mice were anesthetized with sodium pentobarbital (50 mg/kg), photographed, and ultimately euthanized by cervical dislocation. The procedure adhered to the ARRIVE guidelines.

### 2.15 ELISA

The concentration of IL-6 produced by the cell line was detected using the Human IL-6 OneStep ELISA Kit (#S0C3004, STARTER, CHINA). Seed 5 × 10^5^ cells in a 10 cm dish. After cell attachment, replace the complete medium with RPMI 1640 medium. Collect cell supernatants at 6, 24, and 48 hours post-seeding. Dilute the medium threefold before measuring IL-6 concentration.

### 2.16 RELT differentially expressed genes and functional enrichment analysis

Patients with ccRCC were divided into high- and low-expression groups by the median RELT expression. DEGs in the high and low expression groups were differentiated by the ‘Limma’ package of R software using the thresholds of P<0.05 and |Log2(FoldChange)|>1. GO enrichment and KEGG pathway analysis of RELT DEGs were performed using the ClusterProfiler package in addition to GSEA enrichment analysis, and the results were visualized using “ggplot2” visualization. Further weighted correlation network analysis (WGCNA) was performed to find hub genes. Enrichment analysis of hub genes and construction of a least absolute shrinkage and selection operator (LASSO) model. Furthermore, the accuracy of predictive ability was assessed by calculating the AUC of the ROC curve analysis (12, 13). The Spearman correlation between RELT and immune infiltration was examined using methods such as the chi-square test (14).

### 2.17 Protein-protein interaction network

STRING (https://cn.string-db.org/) can be used for searching and constructing PPI interaction networks and can be enriched and analyzed for genes or proteins in the network. The following main parameters are used to create RELT co-expression networks: interaction source; edge; maximum number of interactions; interaction score. In addition, we also input the hub genes into the STRING online platform to construct the PPI network diagram. Use of the TIMER online platform for correlation analysis and visualization of interacting proteins with RELT.

### 2.18 TIMER database

The TIMER (https://timer.cistrome.org/) online data platform includes three modules: Immune Association, Cancer Exploration, and Immune Estimation (15). TIMER can be used to estimate tumor purity, systematic analysis of immune infiltration of various malignancies, and correlation and statistical significance of RELT with immune cells based on various algorithms. The effect of RELT expression versus immune cell expression on survival curves can also be analyzed through the expression module. Expression scatter plots are created by the Co-Expressed Genes Module and are used to assess the correlation and statistical significance of RELT expression with immune cell marker genes.

### 2.19 GEPIA

The GEPIA online database (http://gepia2.cancer-pku.cn/#index) is a TCGA and GTEx database-based 9736 tumor and 8587 normal samples in an integrated network of interactive gene expression analysis (16). The site has seven functional areas for seven main tags: general, differential genes, expression DIY, survival, similar genes, correlation and PCA (16). In this paper, we analyzed the association of RELT expression with various immune cell markers in ccRCC and OS in ccRCC.

### 2.20 Tumor immune single cell hub

The TISCH collects human tumor single-cell RNA-seq datasets from the GEO and ArrayExpress databases, with a total of nearly 2 million cells from 27 cancers in single-cell transcriptome archives (17). In this paper, six ccRCC datasets from TISCH were applied for single-cell transcriptome analysis.

### 2.21 TISIDB

The TISIDB is used to study the interaction between the tumor and the immune system (18). In this study, TISIDB was applied to detect the relationship between RELT and 28 TILs, 45 immune agonists, 24 immunosuppressants, 41 chemokines, and 18 receptors in ccRCC, and a scatter plot was applied for visualization.

### 2.22 Immune cell ratio analysis

The application of the CIBERSORT algorithm is a computational method for estimating the abundance of different cell subpopulations in the tumor microenvironment. It is mainly used to infer the proportion of tumor-infiltrating immune cells (TIICs) from gene expression data. In this paper, the CIBERSORT algorithm was applied to estimate the relative proportions of 22 different TIICs in ccRCC by a linear mixed model. In addition, the ESTIMATE algorithm of the “estimation” package was used to evaluate the StromalScore, ImmuneScore, and ESTIMATEScore of ccRCC samples.

### 2.23 Statistical analyses

In this study, we applied R software (version 4.4.2) to perform all statistical analyses and visualized the results with the help of the ggplot2 software package (version 3.5.1). We applied the Mann-Whitney U-test and paired t-test to explore the potential differences between ccRCC tissues and their neighboring normal tissues. For the results of the K-M curve, GEPIA, and TISIDB analyses, we assessed the TIIC (HR) and P value by log-rank test. To reveal the correlation between RELT expression levels and the degree of immune cell infiltration, immunomodulatory factors, and chemokines, we calculated the Spearman correlation coefficient.

## 3 Results

### 3.1 RELT expression correlates with clinicopathologic features and predicts poor patient prognosis

**Figure 1:**
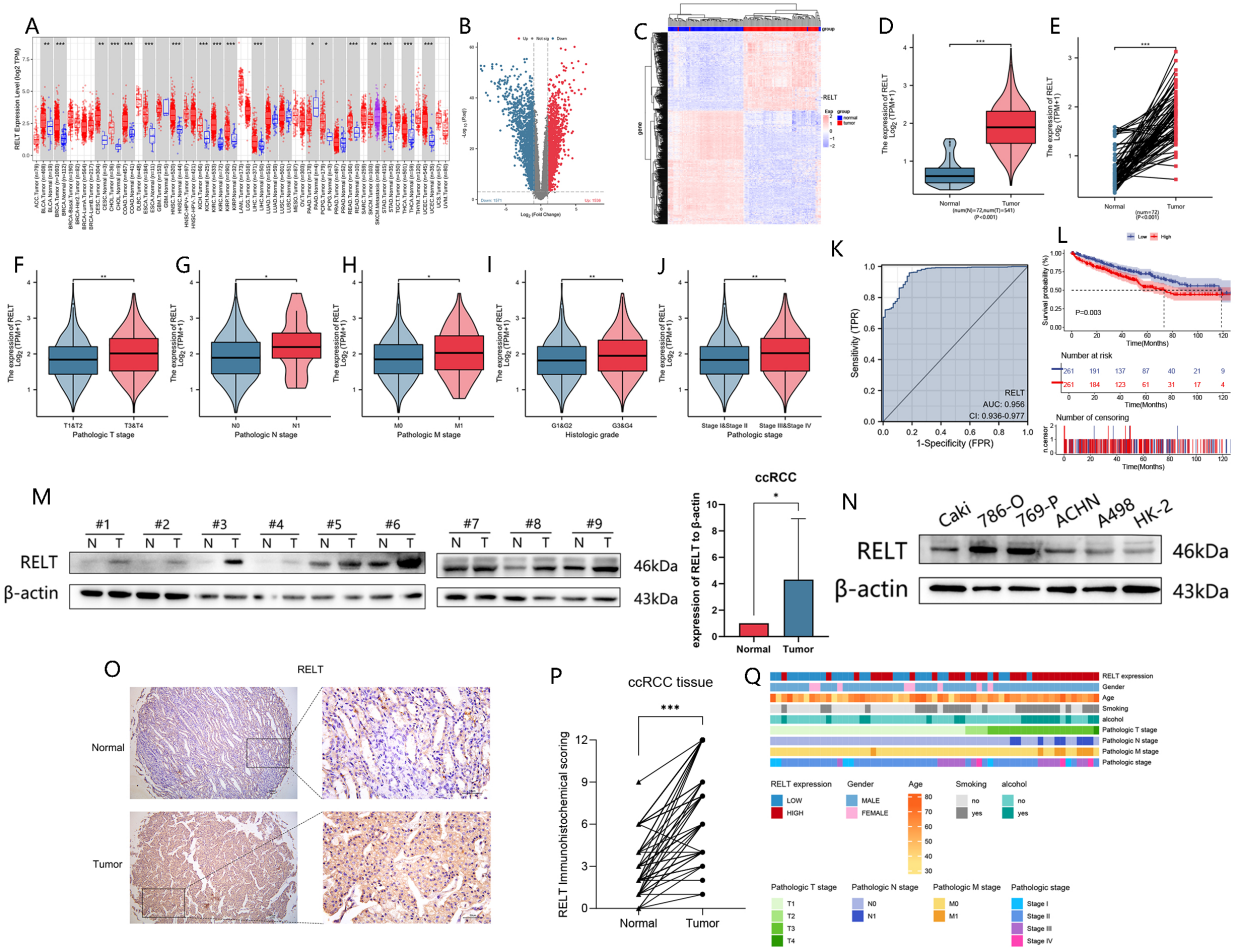
High expression of RELT in ccRCC predicts poor prognosis. **(A)** Increased or decreased RELT in different tumor types from The Cancer Genome Atlas (TCGA) database were identified by Tumor Immunity Estimation Resource (TIMER); **(B)** Analysis of ccRCC samples from the TCGA database, with RELT located in the high-expression group; **(C)** The GEO database of the GSE53757 dataset RELT was highly expressed in the tumor group; **(D)** mRNA expression levels of RELT in 541 ccRCC samples in the TCGA database were higher than in 72 normal samples; **(E)** TCGA database RELT expression levels were high in ccRCC tissues matched to 72 normal tissues; **(F-J)** box plots of RELT expression versus TNM staging **(F-H)**, pathological grading **(I)**, and clinical staging **(J)** in ccRCC samples from the TCGA database; **(K)** diagnostic subject operating characteristic (ROC) curves of RELT in ccRCC; **(L)** Kaplan-Meier of RELT expression versus overall survival; **(M)** RELT expression in ccRCC tissues is higher than that in paracancerous tissues; **(N)** RELT expression in ccRCC cell lines; **(O)** RELT immunohistochemistry experiments of tumors and normal tissues in patients with ccRCC; **(P)** Paired IRS scores for IHC results; **(Q)** Application of component imaging heatmap to visualize the relationship between clinical information and high and low RELT expression in 59 patients; *P<0.05, **P<0.01, ***P<0.001.

According to the whole-cancer analysis of the TCGA database, RELT expression was higher in tumor tissues than in adjacent tissues in 16 types of cancer (**Figure 1A**). To demonstrate the expression levels of RELT at the protein and mRNA levels in ccRCC. First, we analyzed the tumor and adjacent tissues of ccRCC patients from the TCGA database and identified 3,114 differentially expressed genes, of which 1,536 genes were highly expressed in tumors, 1,571 genes were lowly expressed, and RELT was included in the high-expression group (**Figure 1B**). Analyzing the GEO database for visualization, we found that the results were consistent with the previous findings (**Figure 1C**). We conducted an unpaired differential expression analysis between disease and non-disease groups in the TCGA database and found that RELT mRNA expression in ccRCC samples was higher than in normal samples (**Figure 1D**). In addition, paired sample analysis was conducted, revealing that RELT expression in ccRCC tumor samples was higher than in adjacent non-tumor samples (P<0.001) (**Figure 1E**).

**Figures 1F-J**, **Supplementary Figures S1A, B**, and **Table 1** show that high RELT expression correlates with clinical information such as tumor TNM stage, histological grade, pathological grade, patient age, and OS, indicating an unfavorable prognosis. These findings are further supported by pan-cancer OS and disease-specific survival (DSS) analyses (**Supplementary Figure S1C-F**) and Kaplan-Meier survival curve analyses (**Supplementary Figure S1G-K**). The AUC value of the ROC curve for RELT was 0.956 (**Figure 1K**), suggesting that detecting the expression level of RELT in the normal population can be very effective in distinguishing ccRCC patients from the healthy population. In ccRCC, the results of the K-M survival analysis of RELT were consistent with the results of the expression analysis, both suggesting that patients with high expression of RELT have a worse likelihood of survival (**Figure 1L**).

**Table 1:** Correlation of RELT expression with clinicopathological features of ccRCC in the TCGA database.

Characteristics	Low expression of RELT	High expression of RELT	P value
n	270	271	
Pathologic stage, n (%)			0.033
Stage I&Stage II	178 (33.1%)	154 (28.6%)	
Stage III&Stage IV	91 (16.9%)	115 (21.4%)	
Histologic grade, n (%)			0.017
G1&G2	138 (25.9%)	112 (21%)	
G3&G4	127 (23.8%)	156 (29.3%)	
OS event, n (%)			0.023
Alive	195 (36%)	171 (31.6%)	
Dead	75 (13.9%)	100 (18.5%)	
DSS event, n (%)			0.046
No	219 (41.3%)	202 (38.1%)	
Yes	45 (8.5%)	64 (12.1%)	

We extracted proteins from 9 pairs of ccRCC and paracancerous tissues from Yantai Yuhuanding Hospital for Western blot experiments and verified that RELT was highly expressed in ccRCC (**Figure 1M**). And the expression of RELT was verified in the commonly used ccRCC cell lines Caki, 786-O, 769-P, ACHN, A498, and HK-2 (**Figure 1N**), and it was found that RELT expression was highest in 786-O and 769-P. Therefore, we selected two cell lines, 786-O and 769-P, for subsequent cell function experiments. 59 patients from Yantai Yuhuanding Hospital for IHC staining and the IRS for the degree of RELT staining. We found that RELT showed dark brown IHC staining in tumor tissues, indicating that its expression was significantly higher in tumor tissues than in normal tissues (**Figure 1O**). 59 cancer and paracancer paired IRS results were the same as the above findings (**Figure 1P**). We collected clinical data from these 59 patients and applied IRS to divide the patients into a RELT high expression group (IRS > 6) and a RELT low expression group (IRS <= 6). The observed results showed that RELT expression was not correlated with patients’ gender and age but was significantly correlated with patients’ smoking history, alcohol consumption history, TNM stage, and pathologic stage, which was consistent with the results obtained from the bioinformatics analysis above (**Table 2**), and we visualized this result (**Figure 1Q**). Taken together, the above data show that the expression of RELT in ccRCC is significantly elevated, and the expression of RELT is closely related to the clinical data of patients, survival time, and other factors, and it predicts a poor prognosis, and the expression level of RELT in the population can be used to diagnose patients with ccRCC with a high degree of reliability, which suggests that RELT can be used as a new biomarker for the diagnosis of ccRCC.

**Table 2:** Correlation between RELT expression and clinicopathological characteristics of 59 patients in Yantai Yuhuanding Hospital.

Parameters	Number	RELT expression		P value	Statistic	Method
		Low (%)	High (%)			
Patients	59	28	31			
Gender				0.0934	2.816	Chisq test
Male	51	22 (37.29%)	29 (49.15%)			
Female	8	6 (10.17%)	2 (3.39%)			
Age				0.3201	0.9884	Chisq test
≤70	53	24 (40.68%)	29 (49.15%)			
>70	6	4 (6.78%)	2 (3.39%)			
Smoking				0.0227	5.192	Chisq test
yes	26	8 (28.57%)	18 (58.06%)			
no	33	20 (33.90%)	13 (22.03%)			
alcohol				0.0192	5.483	Chisq test
yes	17	4 (6.78%)	13 (22.03%)			
no	42	24 (40.68%)	18 (30.51%)			
Pathologic T stage				0.0025	9.148	Chisq test
T1&T2	39	24 (40.68%)	15 (25.42%)			
T3&T4	20	4 (6.78%)	16 (27.12%)			
Pathologic N stage				0.0038	8.359	Chisq test
N0	51	28 (47.46%)	23 (38.98%)			
N1	8	0 (0.00%)	8 (13.56%)			
Pathologic M stage				0.014	6.033	Chisq test
M0	53	28 (47.46%)	25 (42.37%)			
M1	6	0 (0.00%)	6 (10.17%)			
Pathologic stage				0.0071	7.256	Chisq test
Stage I&Stage II	43	25 (42.37%)	18 (30.51%)			
Stage III&Stage IV	16	3 (5.08%)	13 (22.03%)			

### 3.2 Knock-down RELT affects malignant biological function in ccRCC *in vivo* and *in vitro*

**Figure 2:**
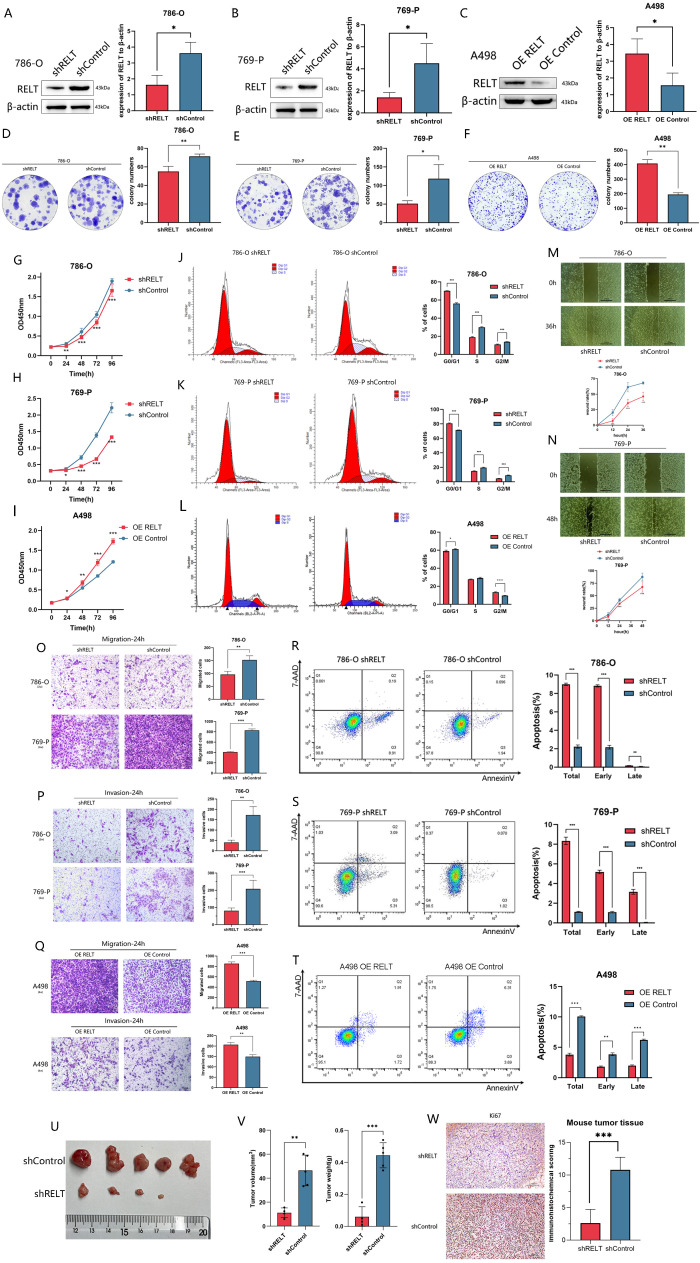
RELT is closely associated with the malignant biological behavior of ccRCC. **(A-C)** Western blot validation of knockdown of RELT in 786-O **(A)** and 769-P **(B)**, overexpressed in A498 cells **(C)**; **(D-F)** Plate cloning assay for proliferation; **(G-I)** CCK-8 assay for proliferation; **(J-L)** Cell cycle analysis; **(M-N)** Wound healing assay for cell migration; **(O-Q)** Transwell migration and Transwell invasion assays for migration/invasion; **(R-T)** Apoptosis assay for cell death; **(U-V)** Nude mouse tumor formation experiments demonstrate RELT gene knockout inhibits tumor growth; **(W)** Application of Ki67 staining in mouse tumor IHC experiments; *P < 0.05, **P < 0.01, ***P < 0.001.

Firstly, according to the above, we selected two cell lines, 786-O and 769-P, and applied shRNA to knock down RELT,or OE RELT overexpression of RELT (**Figures 2A-C**), constructed stable transfected viral cell lines, and performed a series of functional experiments. It was found that knockdown of RELT inhibited the proliferation of 786-O and 769-P,while overexpression of RELT promoted the proliferation of A498 cells. (**Figures 2D-I**). Cell cycle experiments demonstrated that knockdown of RELT could inhibit the G0/G1 to G2/M phase transition of ccRCC cell cycle, whereas RELT overexpression promotes this transition. This mechanism may explain RELT’s influence on cell proliferation (**Figures 2J-L**). Meanwhile, functional assays proved that shRELT could inhibit the migration ability (**Figures 2M-O**) and invasion ability (**Figure 2P**) of renal cancer cells, whereas RELT overexpression promoted invasion and migration of renal cell carcinoma cells (**Figure 2Q**). Flow cytometry demonstrated that decreased RELT expression led to increased apoptosis in ccRCC cells, while overexpression of RELT reduced apoptosis in ccRCC cell lines (**Figures 2R-T**). In addition, we performed tumor formation experiments in nude mice using the 769-P, and we found that the subcutaneous tumor volume and weight of nude mice injected with the ‘shRELT 769-P’ cell line were smaller than those of the control group (**Figures 2U-V**). The mouse tumors were also subjected to IHC experiments to observe the expression of Ki67 in the mouse tumors, and it was found that Ki67 was significantly more highly expressed in the shControl group than in the shRELT group (**Figure 2W**). The above results demonstrate that inhibition of RELT may reduce the malignant biological behavior of ccRCC.

### 3.3 Application of WGCNA analysis and LASSO analysis to explore RELT hub genes

**Figure 3:**
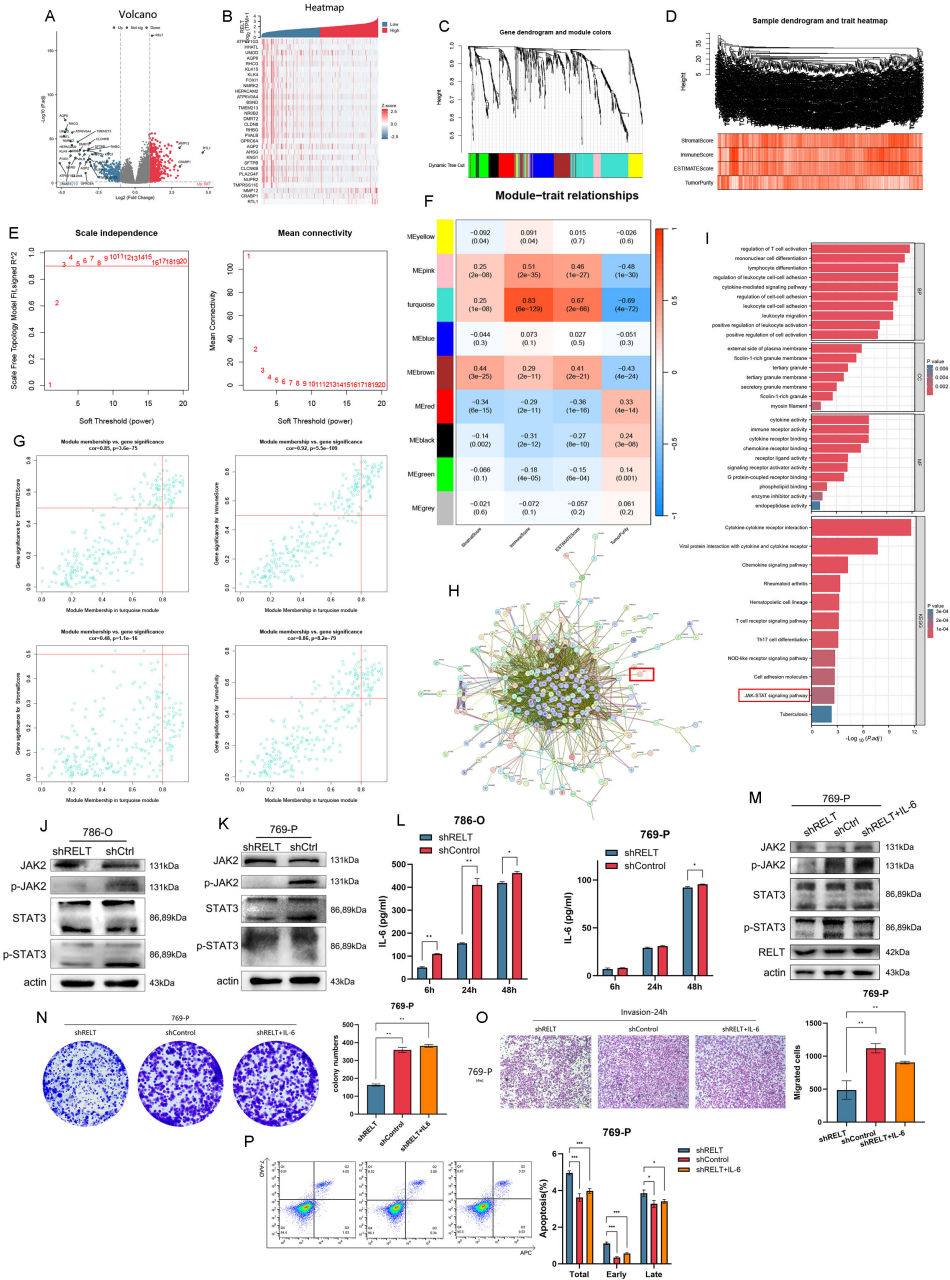
Analysis of RELT hub genes. **(A)** DEGs of RELT in ccRCC; **(B)** Heatmap of the top 30 genes in the DEGs of RELT; **(C)** Clustered module maps of DEGs for WGCNA analysis; **(D)** Dendrograms of sample traits with scores for WGCNA analysis; **(E)** Screening of thresholds for WGCNA module analysis; **(F)** Correlation and P-value of genes with scores for each module of WGCNA analysis; **(G)** Scatter plots demonstrating the gene modules with strong correlation; **(H)** PPI network maps of pivotal genes; **(I)** Pivotal genes with GO and KEGG enrichment analyses; **(J, K)** Western blot validation of the JAK/STAT pathway; **(L)** LASSO coefficients pathway plot and cross-validation curve; **(J, K)** Western blot validation of the JAK2/STAT3 pathway in 786O and 769P cell lines; **(L)** ELISA validation of IL-6 secretion in 786O and 769P cell lines; **(M)** Western blot validation of IL-6 recovery experiments in 769P cell lines; **(N)** Monoclonal assay validation of cell proliferation; **(O)** Transwell migration assay validation of cell migration; **(N)** Flow cytometry analysis of apoptosis.

19,620 genes expressed in ccRCC samples were selected for differential analysis in the TCGA database. According to the high and low RELT expression, they were divided into the high RELT group and the low RELT group and visualized. The log2FoldChange with an absolute value greater than 3 and a p-value less than 0.05 was selected, and DEG analysis between the two groups was found to show 597 up-regulated (red) and 270 down-regulated (blue) differential genes, illustrating the labelled differential genes (**Figure 3A**). The co-expression heatmap showed the top 30 significantly up- and down-regulated genes (**Figure 3B**). Enrichment analysis was performed based on the results of RELT differential gene analysis. GO analysis results indicate that RELT participates in numerous biological processes, including various cellular chemotaxis and humoral immune processes; RELT is mostly expressed in cellular components as protein granules and collagen-containing extracellular matrix and is mostly located on the outer side of the plasma membrane or in the inner lumen of the endoplasmic reticulum; and RELT can function by regulating cytokine activity, peptidase modulator activity, and endopeptidase activity (**Supplementary Figure S1L**). KEGG analysis of RELT revealed that ‘cytokine-cytokine receptor interaction’ was the most important pathway for RELT (**Supplementary Figure S1M**). GSEA analysis of the differential genes showed that RELT achieves its biological functions mainly through substance transport (**Supplementary Figure S1N**).

To construct a WGCNA for RELT, we selected 508 patient samples with complete clinical data from the TCGA database, and for finding the pivotal genes, we performed the WGCNA analysis on the basis of 867 differential genes. After a series of adjustments to the WGCNA parameters, the DEGs were classified into 9 modules by average linkage hierarchical clustering (**Figures 3C-E**). The genes of the turquoise module were selected as hub genes (absolute module affiliation [MM] > 0.5, absolute gene significance [GS] > 0.5) (**Figure 3F**). The turquoise module in **Figure 3F** contained 265 genes and had the highest correlation with the ImmuneScore (Pearson correlation coefficient = 0.83, P < 0.0001) and TumorPurity (Pearson correlation coefficient = -0.69, P < 0.0001) of RELT (**Figure 3G**). We extracted the genes of the turquoise module to construct the PPI network (**Figure 3H**) and applied GO and KEGG analysis to analyze the hub genes of RELT. GO analysis showed that RELT was associated with the regulation of immune cells; RELT was mostly manifested as protein granules, which was the same as the result of the 867 differential gene enrichment analysis, and RELT played a role in the regulation of cytokine activity and regulation of enzyme activity, etc. KEGG analysis indicated that RELT might be strongly correlated with the JAK-STAT signaling pathway (**Figure 3I**). We validated this by applying Western blot experiments in two cell lines, 786-O and 769-P, and found that knockdown of RELT reduced the activation of the JAK/STAT pathway (**Figures 3J, K**).

Additionally, we added the STAT3/JAK2 pathway agonist IL-6 to the RELT-knockdown 769-P cell line (19), revealing that IL-6 can reverse the inhibition of the STAT3/JAK2 pathway caused by RELT knockdown. Beyond validation via Western blot experiments (**Figure 3L**), we conducted cell proliferation assays, Transwell migration assays, and apoptosis assays, further enhancing the credibility of the results. The above experimental results demonstrate that the STAT3/JAK2 pathway agonist IL-6 reverses the inhibition of cell proliferation and migration caused by RELT knockdown and also reverses the promotion of apoptosis (**Figures 3M-O**).

### 3.4 LASSO regression analysis

**Figure 4:**
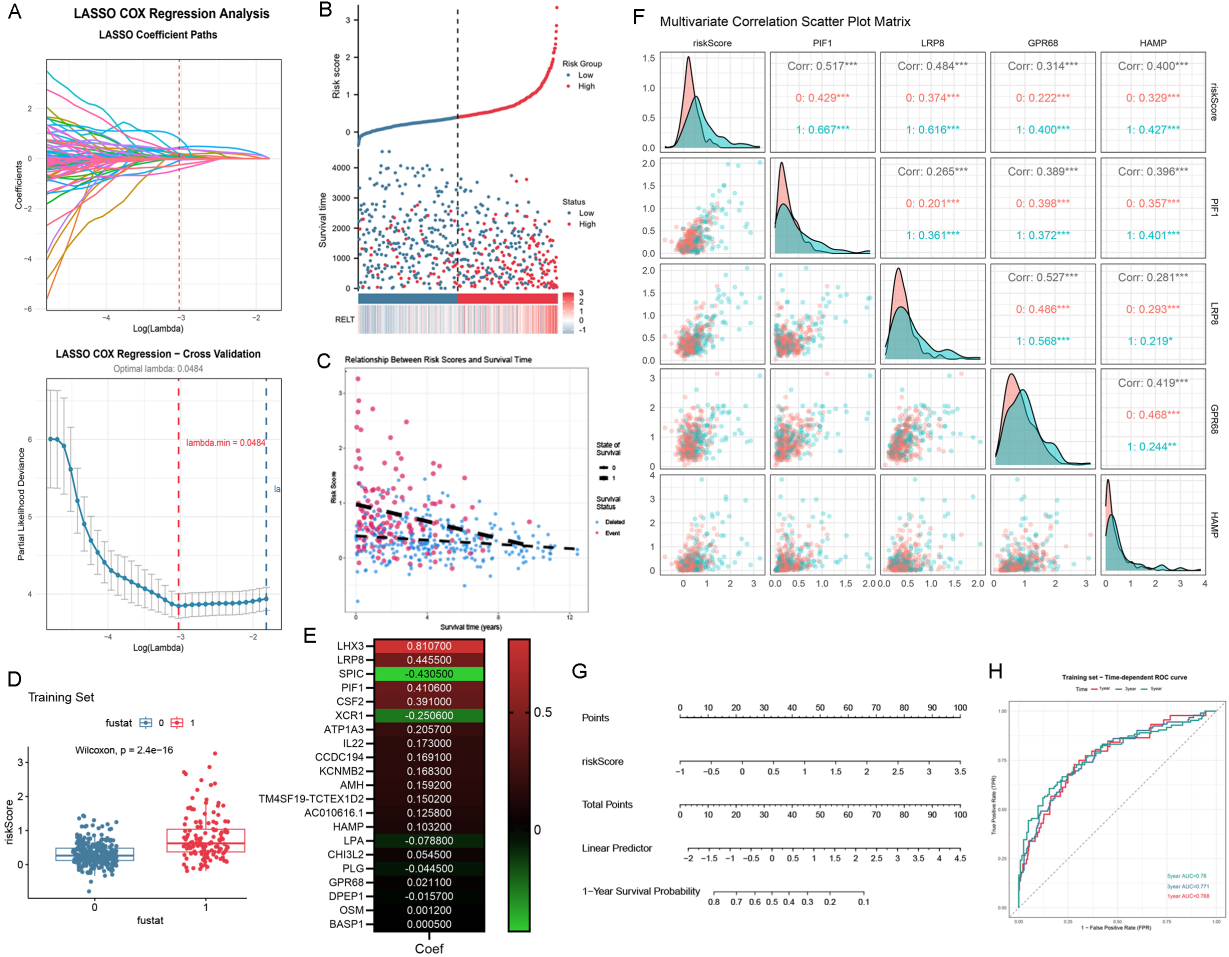
LASSO regression analysis. **(A)** Path diagram of regression coefficients from LASSO regression and cross-validation curve diagram; **(B)** RELT expression distribution, survival status and RELT expression profiles, with blue dots representing surviving ccRCC patients and red dots representing dead ccRCC patients; **(C)** Relationship between risk scores and survival time in the training set; **(D)** Risk score versus survival boxplot for the training set, where 1 indicates a death event occurred and 0 indicates no death event occurred; **(E)** Heatmap of 21 Genes and Regression Coefficients in the Predictive Model; **(F)** Multivariate correlation scatter plot matrix of four highly correlated genes; **(G)** Prognostic model prognostic nomogram; **(H)** The ROC curve for predictive accuracy showed an AUC value of 0.78 at 5 years, 0.771 at 3 years, and 0.768 at 1 year.

We performed a LASSO regression analysis of the genes in the turquoise module and constructed a prognostic prediction model (**Figures 4A, B**). The dataset was split into a 30% test set and a 70% training set, and cross-validation was performed. In the prognostic model, we found that the survival status of ccRCC patients, the distribution of RELT expression, and the expression profile of RELT increased with the increase of the risk factor scores of ccRCC patients, and the number of deaths of ccRCC patients increased accordingly (**Figures 4B-D**). The prediction model we built included 21 genes, and their regression coefficients are shown in **Figure 4E**. We visualized the four highly correlated genes—PIF1, LRP8, GPR68, and HAMP—using a Multivariate Correlation Scatter Plot Matrix (**Figure 4F**). Based on this prognostic model, we visualized the prognostic nomogram (**Figure 4G**). This model demonstrates that higher risk scores, higher points, and higher linear predictors correlate with lower 1-year survival probability. Additionally, we plotted the model’s ROC curve, yielding an AUC of 0.768 at 1 year, 0.771 at 3 years, and 0.780 at 5 years (**Figure 4H**). Due to the small sample size of the validation set, the generated calibration curve confidence intervals are excessively wide, resulting in unstable and unreliable evaluation outcomes. Therefore, the calibration curve has not been visualized.

In addition, in the STRING database, we constructed an interaction network of RELT with 10 co-expressed genes, and based on this, we constructed a PPI network (**Supplementary Figure S1O**). The expression level of RELT was positively correlated with immune-related genes such as TRAF1 (tumor necrosis factor receptor-associated factor 1), RELL2, and TNFRSF4 (**Supplementary Figure S1P**). Existing reports demonstrated that TRAF1 is a signaling mediator for members of the TNFRSF, which regulate a variety of signaling pathways, including the MAPK, NF-κB, and Akt pathways, which play key roles in immune cell activation, inflammatory response, and cell survival (20). RELL2 is poorly associated with the prognosis of several cancers and is strongly associated with the expression of immune checkpoints (21). TNFRSF4 (OX40), a member of the TNFRSF, interacts with TNFRSF4L (OX40L), which is selectively expressed on Tregs, which are regulatory T cells that have the function of regulating immune responses (22). All of these molecules have been linked to immunity, which provides more solid evidence for the use of RELT as a biological marker associated with immune infiltration.

### 3.5 RELT is strongly associated with immune infiltration

**Figure 5:**
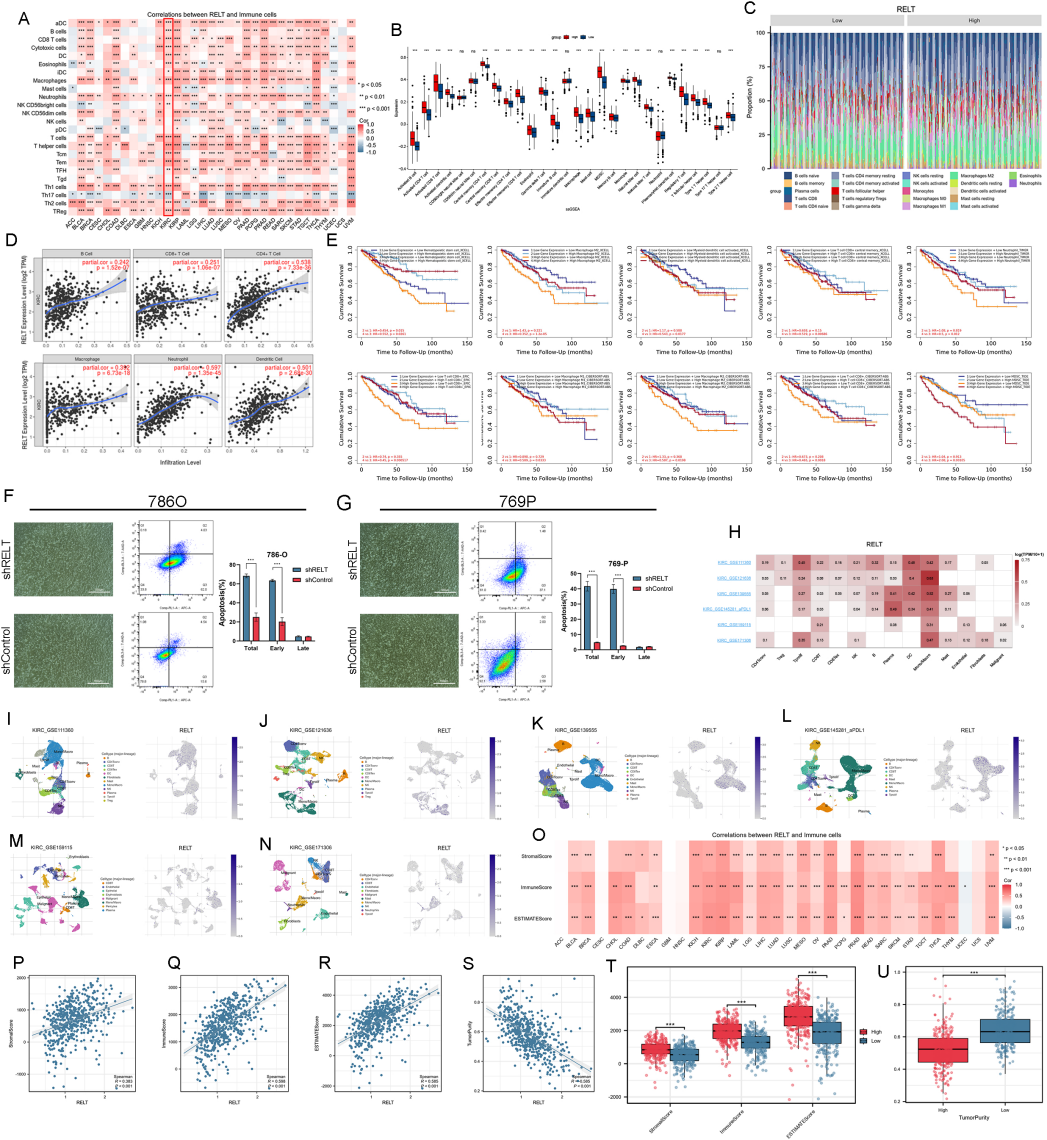
Immune infiltration analysis of RELT in ccRCC. **(A)** Correlation analysis of RELT with immune cells in pan-cancer under the ssGSEA algorithm; **(B)** Unpaired box plots of RELT high and low expression subgroups versus immune cell expression under the ssGSEA algorithm; **(C)** Plot of the ratio of high and low expression of RELT versus immune cells; **(D)** Scatter plots of correlation between RELT expression and immune cells; **(E)** K-M curves of the prognostic impact of high and low RELT expression as well as high and low immune cell expression on patient prognosis; **(F)** Brightfield image of T cells co-cultured with tumor cells; **(G)** Analysis of Tumor Cell Apoptosis in Co-culture with T Cells; **(H)** Correlation of RELT expression with immune cells in ccRCC dataset of TISCH online platform; **(I-N)** Expression of RELT in ccRCC single-cell dataset of TISCH online platform; **(O)** Correlation of RELT expression with immune cell score; **(P-S)** Scatter plots of RELT expression versus StromalScore **(P)**, ImmuneScore **(Q)**, ESTIMATEScore **(R)**, and TumorPurity **(S)**; **(T)** Box plots of the relationship between high and low groupings of RELT expression and scores; **(U)** Box plots of the relationship between high and low groupings of RELT expression and tumor purity; *P<0.05, **P<0.01, ***P<0.001.

In the TIMER database, RELT was clearly correlated with most immune cell markers, including T cells, B cells, monocytes, and macrophages (**Table 3**), and the RELT immune cell marker analyses in the GEPIA public database had the same results (**Table 4**). In order to analyze the correlation between RELT expression and immune cell infiltration in ccRCC, we applied various algorithms such as ssGSEA, CIBERSORT, EPIC, TIMER, and xCell in pan-cancer. Genetic techniques used to study immune infiltration had to take into account the purity of tumor cells in clinical cancer samples and the expression of RELT versus ccRCC (r=-0.321, p<0.001) purity (**Supplementary Figure S1Q**). Immune cell correlation analysis showed that RELT expression was positively correlated with T helper cells (r=0.476, p<0.001), Tem (r=0.443, p<0.001), and macrophages (r=0.340, p<0.01), and negatively correlated with Th17 cells (r=-0.296, p<0.001) (**Supplementary Figure S1R** & **5A**). TReg cells in cancer may promote tumor escape by suppressing anti-tumor immune responses (23). Macrophages are part of the immune system and play a role in promoting the progression of tumors (24).The role of Th17 cells in cancer is not fully understood, but some studies have shown that they may contribute to the development of cancer by promoting inflammation and angiogenesis (23). In ccRCC, an increase in TH17 cells was associated with significantly improved survival (25). We therefore suggest that RELT may play a role in promoting immune escape in ccRCC. We carried out an analysis of the role of RELT in the ccRCC tumor immune microenvironment based on multiple algorithms. First, the ssGSEA algorithm was applied to analyze the correlation between RELT expression and immune cells, and it was found that RELT expression was positively correlated with a variety of immune cells in pan-cellular carcinomas (**Figure 5A**). Unpaired box plot visualization based on the correlation between RELT expression and the abundance of 28 immune cells analyzed by this algorithm revealed that RELT expression was significantly and positively correlated with the abundance of 23 of these immune cells in ccRCC, and the results of the analysis showed that the enriched fraction of immune cells was higher in the group with high RELT expression (**Figure 5B**). In the immune cell proportion analysis, macrophages M2 were significantly correlated with RELT expression, and T cell regulatory Tregs were proportionally increased in the RELT high expression group (**Figure 5C**). The results of RELT expression and immune cell correlation analyses on the TIMER public platform showed a significant correlation with B cell (r=0.242), CD8+ T cell (r=0.251), CD4+ T cell (r=0.538), macrophage (r=0.392), neutrophil (r=0.597), and dendritic (r=0.501) cells, all of which showed a significant positive correlation (**Figure 5D**). Application of CIBERSORT, EPIC, TIMER, and xCell algorithms for more immune cell correlation analyses revealed that RELT was associated with T cells, follicular Tregs, activated dendritic cells, resting mast cells, cancer-associated fibroblasts, macrophages, T cell CD8+, myeloid dendritic cells, T cell CD4+, and neutrophils (**Supplementary Figures S1S, T**).

**Table 3:** Correlation analysis of RELT with immune cell-related genes and markers in the Tumor Immunity Evaluation Resource (TIMER2.0).

Description	Gene markers	ccRCC none cor	P	Purity cor	P
CD8 T cell+	CD8A	0.377	***	0.314	***
	CD8B	0.344	***	0.288	***
	TBX21	0.413	***	0.386	***
	IFNG	0.401	***	0.330	***
	CXCL9	0.394	***	0.324	***
	CXCL10	0.329	***	0.261	***
T cell(general)	CD3D	0.411	***	0.336	***
	CD3E	0.436	***	0.362	***
	CD3G	0.421	***	0.366	***
	CD2	0.420	***	0.344	***
B cell	CD19	0.419	***	0.362	***
	CD79A	0.377	***	0.314	***
	BLK	0.432	***	0.377	***
Monocyte	CD86	0.528	***	0.482	***
	CD115(CSF1R)	0.581	***	0.541	***
TAM	CCL2	0.188	***	0.138	**
	CD68	0.303	***	0.311	***
	IL10	0.466	***	0.403	***
	CSF2	0.319	***	0.314	***
M1 Macrophage	INOS(NOS2)	0.166	***	0.124	**
	IRF5	0.364	***	0.379	***
	COX2(PTGS2)	0.233	***	0.183	***
M2 Macrophage	CD163	0.450	***	0.424	***
	VSIG4	0.479	***	0.433	***
	MS4A4A	0.463	***	0.417	***
Neutrophils	CD66b(CEACAM8)	0.125	**	0.142	**
	CD11b(ITGAM)	0.541	***	0.501	***
	CCR7	0.471	***	0.425	***
Netural killer cell	KIR2DL1	0.106	*	0.054	0.246
	KIR2DL3	0.095	*	0.074	0.111
	KIR2DL4	0.231	***	0.190	***
	KIR3DL1	0.053	0.221	0.047	0.312
	KIR3DL2	0.128	**	0.119	*
	KIR3DL3	0.164	***	0.147	**
	KIR2DS4	0.118	**	0.093	*
Dendritic cell	HLA-DPB1	0.430	***	0.430	***
	HLA-DQB1	0.315	***	0.315	***
	HLA-DRA	0.392	***	0.392	***
	HLA-DPA1	0.386	***	0.386	***
	BDCA-1(CD1C)	0.248	***	0.248	***
	BDCA-4(NRP1)	0.160	***	0.160	***
	CD11c(ITGAX)	0.623	***	0.623	***
Th1	T-bet(TBX21)	0.413	***	0.413	***
	STAT4	0.479	***	0.479	***
	STAT1	0.422	***	0.422	***
	IFN-γ(IFNG)	0.401	***	0.401	***
	TNF-α(TNF)	0.376	***	0.376	***
Th2	GATA3	0.217	***	0.217	***
	STAT6	0.187	***	0.187	***
	STAT5A	0.495	***	0.495	***
	IL13	0.251	***	0.251	***
Tfh	BCL6	0.270	***	0.270	***
	IL21	0.204	***	0.204	***
Th17	STAT3	0.336	***	0.336	***
	IL17A	0.047	0.276	0.047	0.276
Treg	FOXP3	0.491	***	0.491	***
	CCR8	0.427	***	0.427	***
	STAT5B	0.106	**	0.106	**
	TGFβ(TGFB1)	0.346	***	0.346	***
T cell exhaustion	PD-1(PDCD1)	0.460	***	0.460	***
	PD-L1(CD274)	0.233	***	0.233	***
	CTLA4	0.502	***	0.502	***
	LAG3	0.456	***	0.456	***
	TIM-3(HAVCR2)	0.100	*	0.100	*
	GZMB	0.342	***	0.342	***

The symbols *, **, and *** denote the P-values of the correlation coefficients for the corresponding genes.

**Table 4:** Correlation analysis of RELT with monocyte, macrophage, and T-cell depletion-related genes and markers in Gene Expression Profiling Interaction Analysis (GEPIA).

Description	Gene markers	ccRCC Tumor cor	P	Normal cor	P
CD8 T cell+	CD8A	0.33	***	0.63	***
	CD8B	0.29	***	0.49	***
	TBX21	0.38	***	0.52	***
	IFNG	0.37	***	0.34	**
	CXCL9	0.35	***	0.28	*
	CXCL10	0.24	***	0.35	**
T cell(general)	CD3D	0.34	***	0.63	***
	CD3E	0.4	***	0.71	***
	CD3G	0.39	***	0.72	***
	CD2	0.36	***	0.67	***
B cell	CD19	0.29	***	0.58	***
	CD79A	0.22	***	0.55	***
	BLK	0.3	***	0.62	***
Monocyte	CD86	0.49	***	0.68	***
	CD115(CSF1R)	0.58	***	0.62	***
TAM	CCL2	0.11	*	0.69	***
	CD68	0.29	***	0.53	***
	IL10	0.41	***	0.44	***
	CSF2	0.29	***	0.39	***
M1 Macrophage	INOS(NOS2)	0.074	0.091	0.25	*
	IRF5	0.28	***	-0.077	0.520
	COX2(PTGS2)	0.057	0.190	0.21	0.083
M2 Macrophage	CD163	0.48	***	0.61	***
	VSIG4	0.48	***	0.63	***
	MS4A4A	0.46	***	0.61	***
T cell exhaustion	PD-1(PDCD1)	0.34	***	0.55	***
	PD-L1(CD274)	0.2	***	0.44	***
	CTLA4	0.44	***	0.54	***
	TIM-3(HAVCR2)	0.026	0.550	0.43	***
	GZMB	0.32	***	0.67	***

The symbols *, **, and *** denote the P-values of the correlation coefficients for the corresponding genes.

K-M curves between high and low RELT expression groups and between high and low immune cell groups were analyzed according to the five algorithms mentioned above on the TIMER public online platform. It was found that there was basically no difference in the immune cells in the low expression RELT group. Whereas in the high expression RELT group, there was a difference between the group with low hematopoietic stem cells, macrophage M1, macrophage M2, myeloid dendritic cell activated, T cell CD8+ central memory, T cell CD8, and neutrophil’s group and the group with higher MDSC that tended to be accompanied by a worse prognosis, we suggest that RELT may enhance immune escape from ccRCC by inhibiting T cells and promoting suppression of the immune response to MDSC (**Figure 5E**). Following co-culture of the 786O-shRELT, 786O-shCtrl, 769P-shRELT, and 769P-shCtrl cell lines with T cells, we detected increased apoptosis in tumor cells following RELT knockdown. This further demonstrates that RELT may exert an inhibitory effect on T cells, thereby promoting immune escape in ccRCC (**Figures 5F-G**). We performed the analysis based on the following datasets from the TISCH open platform (**Figures 5H-N**). We aimed at understanding the expression pattern of RELT in ccRCC. In the above ccRCC dataset, all of them showed that RELT was expressed in monocytes or macrophages, which was consistent with the results of the previous analyses and enhanced the credibility of this result. Taken together with the above results of immune cell infiltration analysis, we believe that RELT expression correlates strongly with immune cells such as CD4 T cells, CD8 T cells, T helper cells, macrophages, neutrophils, and DCs, which all prove that RELT is closely related to immune escape from ccRCC.

Immunoscore as well as tumor purity analyses based on data from the TCGA database found that RELT was positively correlated with both StromalScore, ImmuneScore, and ESTIMATEScore in multiple cancers (**Figure 5O**). Analysis of the correlation between RELT expression and scores in ccRCC found that RELT was positively correlated with all three scores and negatively correlated with tumor purity (**Figures 5P-S**). The higher the RELT expression in ccRCC, the higher the StromalScore, ImmuneScore, and ESTIMATEScore, and the lower the TumorPurity (**Figures 5T-U**). It is well known that low tumor purity tends to be independently associated with shorter survival time and faster recurrence (26). This implies that higher RELT expression and lower tumor purity in ccRCC predict a poorer prognosis and a higher likelihood of recurrence in ccRCC patients.

### 3.6 RELT is associated with immunomodulators and chemokines

**Figure 6:**
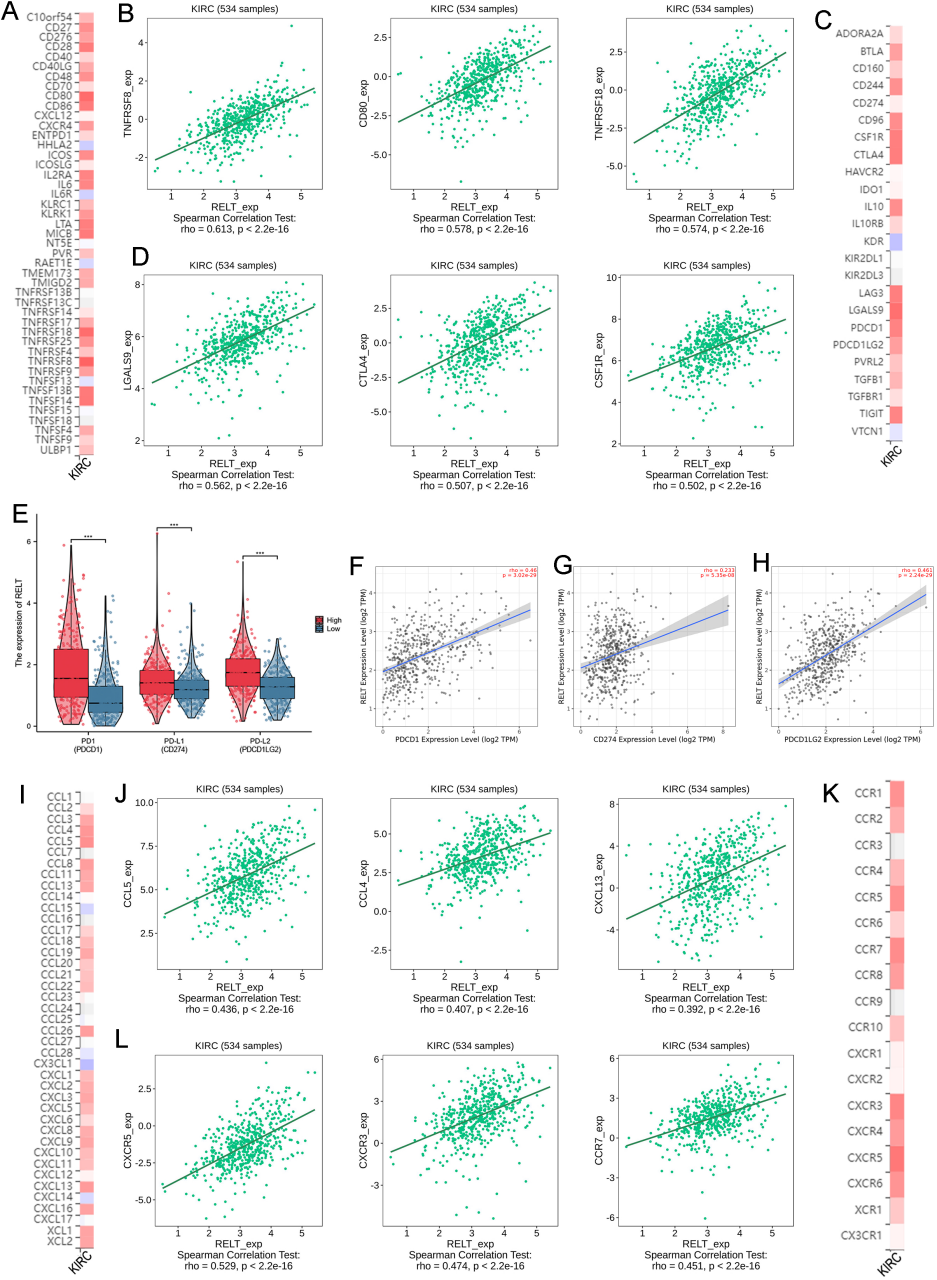
RELT in relation to immunomodulators, chemokines, and chemokine receptors. **(A, B)** Heatmap and scatterplot of correlation between RELT expression and multiple immune agonists; **(C, D)** Heatmap and scatterplot of correlation between RELT expression and multiple immunosuppressants; **(E)** Box plots of RELT expression in high and low subgroups versus the expression of the three major star checkpoints; **(F-H)** Scatterplot of correlation between the three major immune checkpoints and RELT expression; **(I, J)** Heatmap and scatterplot of correlation between RELT expression and multiple chemokines; **(K, L)** Heatmap and scatterplot of correlation between RELT expression and multiple chemokine receptors; *P<0.05, **P<0.01, ***P<0.001.

In the correlation analysis of RELT with immunomodulators, RELT was found to be positively correlated with both multiple immunoagonists (**Figures 6A, B**) and multiple immunosuppressants (**Figures 6C, D**). Correlation scatter plots are shown in **Supplementary Figure S2A** and **Supplementary Figure S2B**. Visualizing the differences in the expression of the three major star immune checkpoints PD-1, PD-L1, and PD-L2 between the high and low RELT groups, it was found that the expression of PD1, PD-L1, and PD-L2 was higher in the high RELT expression group compared to that in the low RELT expression group (**Figure 6E**). Visualisation of RELT expression levels based on PD-1, PD-L1, and PD-L2 expression levels in visualized scatter plots revealed that RELT and all three immune checkpoints were positively correlated (**Figures 6F-H**). Rooted in the results of the above analysis, RELT may function in immune regulation and may be involved in tumor immune escape by regulating PD1 and PD-L1.

In addition to this, chemokines play an important role in tumor immune escape. By analyzing the correlation between RELT expression and chemokines and their receptors, we found that RELT expression was significantly and positively correlated with a variety of chemokines (**Figures 6I, J**) and with a variety of chemokine receptors (**Figures 6K, L**). Scatter plots of the correlation of RELT with a wider range of chemokines and their receptors are shown in **Supplementary Figure S2C** and **Supplementary Figure S2D**. These analyses suggest that RELT may play a role as an immunomodulatory factor in ccRCC.

## 4 Discussion

ccRCC is a common urological tumor with an insidious onset, often detected incidentally, and many patients with ccRCC are diagnosed in the absence of obvious symptoms (4). ccRCC is insensitive to conventional chemotherapy but responsive to targeted therapy and immunotherapy (27). ccRCC has an abundant T-cell infiltrate, which renders it potentially responsive to immune checkpoint-blocking therapies (27). Approximately 30% of patients with ccRCC who have undergone radical nephrectomy eventually develop metastatic disease (4). Metastatic renal cell carcinoma (mRCC) has also traditionally been insensitive to conventional radiotherapy and chemotherapy, and immunotherapeutic agents such as interferon-α and/or interleukin-2 were once the treatment of choice (28). In summary, the treatment of ccRCC remains a major challenge, but ccRCC is characterized by abundant T-cell infiltration, which makes immunotherapy a potential direction for the treatment of ccRCC (29). Because of the heavy difficulties encountered in the treatment of ccRCC, there is an urgent need to discover a new biomarker for the early diagnosis and treatment of ccRCC.

RELT, also known as Receptor Expressed in Lymphoid Tissues, belongs to one of the members of the TNFRSF, a group of cell-surface receptors that bind to ligands of the TNF superfamily (e.g., LIGHT, LTα, etc.) and are involved in the regulation of T-cell activation, proliferation, differentiation, and survival (30). Also, RELT is a protein expressed in lymphoid tissues (21), which are expressed in lymphoid and haematopoietic tissues (31). Usually RELT is considered an independent receptor whose ligand has not been identified, and its exact biological function and role in disease are still under investigation. In the study of RELT and cancer, we found that RELT was associated with immune infiltration in a variety of cancers, including ESCC, PC (prostate cancer), HCC (hepatocellular carcinoma), lung adenocarcinoma, colorectal cancer, and gastric cancer (10). It has been shown that the elevated expression level of RELT in ESCC promotes the malignant biological behavior of ESCC (9). Although RELT has been shown to be associated with T cell-mediated immune infiltration (10), the relationship between RELT expression and ccRCC immune infiltration is unclear.

In this paper, we systematically investigated the expression level of RELT in ccRCC and its clinical significance. Our analyses showed that high RELT expression predicted a poor prognosis for ccRCC. Furthermore, RELT expression in ccRCC correlated with the degree of infiltration of immune cells, immunomodulators, chemokines, and receptors, suggesting the possibility of RELT as a molecule targeted for immunotherapy. We assessed the mRNA level and protein level expression of RELT in ccRCC by online databases such as TCGA, GEO, and GEPIA, which was significantly higher in ccRCC tissues than in paracancerous tissues. We analyzed the association between RELT expression and patients’ clinical profiles in the ccRCC cohort using the GEPIA, K-M, and TIMER online platforms and the TCGA database. The analysis found that higher levels of RELT expression in ccRCC were often accompanied by higher TNM stage, clinical stage, pathological grade, and poorer OS and DSS, and the results of the survival analysis were consistent with the previous results. Based on LASSO regression analysis, ROC curves, and prognostic column line plots, RELT can be used as a biomarker for predicting prognosis with a high level of confidence. Among the 21 genes in the predictive model, the four most strongly correlated genes—PIF1, LRP8, GPR68, and HAMP—include GPR68, an acid-sensing G protein-coupled receptor activated in acidic environments. This receptor is closely associated with the Warburg effect, tumor progression, and treatment resistance (32). Following the establishment of stable shRNA-viral transfections in 786-O and 769-P cell lines, a series of functional experiments revealed that RELT knockdown mitigates the malignant biological behavior of clear cell renal cell carcinoma (ccRCC), whereas RELT overexpression promotes ccRCC progression.

Regarding the mechanism by which RELT promotes immune evasion in ccRCC, we conducted *in vitro* experiments based on enrichment analysis results. The findings demonstrated that RELT knockdown reduced IL-6 secretion by ccRCC cells, thereby decreasing JAK2/STAT3 pathway activity. Exogenous IL-6 restored the impaired proliferation, migration, and increased apoptosis induced by RELT knockdown in ccRCC cells. IL-6 is a well-known specific agonist of the JAK2/STAT3 pathway (19, 33), leading us to conclude that RELT regulates this pathway upstream. Consistent with the notion that the IL-2 cytokine binds to T-cell surface receptors to specifically activate the downstream JAK/STAT pathway (34), we propose that IL-6 may exert a similar effect on. Notably, studies have demonstrated that activation of the JAK/STAT pathway influences T cell developmental differentiation and modulates key lymphocyte effector functions (35), suggesting RELT exerts significant and profound effects on T cell developmental pathways. The cytotoxic capacity of T cells is not suddenly acquired upon antigen exposure but rather is a core potential that is pre-programmed and strictly regulated during their thymic development. This perspective clearly indicates that the developmental process of T cells is closely linked to their cytotoxic capabilities (36). Our *in vitro* experiments co-cultured tumor cells with T cells, demonstrating that RELT knockdown significantly enhanced T cell-mediated killing of ccRCC cells, leading to increased apoptosis. This finding further substantiates that RELT may influence T cell development, thereby promoting immune escape and diminishing T cell anti-tumor activity. Furthermore, as demonstrated by Chang et al., TGF-β and IL-6 frequently exert synergistic effects (37), with their signaling pathways often exhibiting crosstalk to form cooperative inhibitory networks. Given that TGF-β is a key factor suppressing NK cell function (38), we hypothesize that RELT may also suppress NK cell activity in ccRCC by interacting synergistically with TGF-β within the inflammatory environment created by IL-6. The function and migration of NK cells exhibit circadian rhythmicity. TGF-β and IL-6, influenced by circadian rhythms, are prone to dysregulation within the tumor immune microenvironment (39). In future studies, we will conduct more in-depth investigations by establishing a periodic dosing model that simulates circadian disruption (40). These findings collectively provide compelling evidence of RELT’s impact on the ccRCC tumor immune microenvironment, establishing RELT as a viable prognostic indicator for ccRCC.

In tumor immunity, cellular immunity occupies a major position and plays a crucial role. RELT not only binds selectively to TRAF1 but also stimulates T-cell proliferation and regulates immune responses in the presence of CD3 signaling, as well as regulating inflammation, cell proliferation, apoptosis, and morphogenesis (41, 42). Several articles have indicated that TNFRSF (including RELT, RELL1, and RELL2) can play a role in influencing immune infiltration through the NF-κB pathway (8–10, 43, 44). In addition, humoral immunity plays an important synergistic role in the tumor immune response. Co-staining of RELT with CD20 indicates that RELT is expressed in malignant B cells, and it has been shown that RELT may be associated with B-cell lymphoma development and progression (45). Based on the above research basis, this article systematically investigated the correlation between RELT expression in ccRCC and immune infiltration. Firstly, we performed a correlation analysis, and we found that RELT expression was closely associated with immune cells such as TIL, including T helper cells, CD8 T cells, CD4 T cells, macrophages, T cells, Th1 cells, macrophages, neutrophils, and DC. And high RELT expression was positively correlated with StromalScore, ImmuneScore and ESTIMATEScore. This means that high RELT expression is often accompanied by high levels of immune cell infiltration, stromal cell infiltration, and lower tumor purity, which implies that ccRCC patients with high RELT expression may have shorter OS and faster recurrence rates. In addition, RELT correlates with a variety of immunomodulatory factors and chemokines and their receptors. We systematically analyzed the correlation of RELT with immune cell-related marker genes in ccRCC. We found that RELT correlates extremely well with macrophages. Macrophages are an important component of the innate and adaptive immune system, and they play a role in fighting pathogens and regulating organismal homeostasis (46). Macrophages promote tumor progression (47), angiogenesis, and systemic spread by promoting genetic instability, nourishing cancer stem cells, supporting metastasis, and suppressing protective adaptive immunity (48). While CD163, VSIG4, and MS4A4A are markers of macrophages. CD163 is a transmembrane scavenger receptor expressed on the surface of macrophages, which plays an important role in macrophage activation and function (49). Macrophages expressing CD163 are associated with poor prognosis, reduced overall survival, and metastasis in a variety of malignancies (49). VSIG4 is a type I transmembrane receptor that is exclusively expressed on the surface of macrophages (50). VSIG4-positive macrophages in the tumor microenvironment may promote cancer progression and correlate with low CD8+ T-cell frequency, high Foxp3+/CD8+ infiltrating T-cell ratios, and poor prognosis (50). MS4A4A promotes the polarization of M2-type macrophages through activation of the PI3K/AKT and JAK/STAT6 signaling pathways (51). The MS4A4A-positive macrophages in the tumor microenvironment correlate with CD8+ T cell dysfunction, which may contribute to tumor immune escape (51). In contrast, RELT correlated with macrophage markers CD163, VSIG4, and MS4A4A when analyzed by applying the TIMER and GEPIA online platforms. All of the above analyses demonstrated a significant correlation between RELT and ccRCC immune escape.

Our study still has limitations. First, the data in this paper has limitations; most of them come from the databases of online platforms, without obtaining the latest information about clinical samples. Furthermore, the analysis of single-cell transcriptomic data is currently limited to the expression stage. In future studies, we will conduct more in-depth analyses by following comprehensive single-cell transcriptomic data analysis guidelines (52). Furthermore, due to the limited sample size of the validation set, we were unable to evaluate model performance metrics such as the C-index and calibration plots. Finally, our study applies bioinformatics analysis and some ex vivo and *in vivo* experiments to analyze the function and mechanism of RELT, but *in vivo* and ex vivo experimental validation of RELT exerting its function is necessary.

## 5 Conclusion

In this study, we determined that high expression of RELT in ccRCC predicts its poor prognosis and is strongly associated with the tumor immune microenvironment. The analyses suggest that RELT may act as an immune infiltration-associated molecule to predict the prognosis of ccRCC. However, the mechanism of how RELT promotes ccRCC development remains to be further explored.

## Data Availability

The original contributions presented in the study are included in the article/**Supplementary Material**. Further inquiries can be directed to the corresponding author/s.

## Supplementary material

Supplementary Figure 1 Supplementary analysis on the role, relevance, and immune infiltration of RELT with respect to prognosis. **(A)** Forest plot of RELT overall survival in pan-cancer; **(B, C)** RELT expression was associated with clinical forest plots and grouped image heatmaps of pathological characteristics; **(D)** relationship between RELT expression and overall survival in pan-cancer; **(E)** box plot of RELT expression versus overall survival in ccRCC; **(F)** box plot of RELT expression versus DSS in ccRCC; **(G)** high and low RELT expression within the T3, T4, N0, and M1 stages of ccRCC affecting the prognosis of the patients’ K-M curves; **(H)** Relationship between RELT expression and histological grading in pan-cancer; **(I)** K-M curves of high and low RELT expression within G4 grading of ccRCC affecting patients’ prognosis; **(J)** Relationship between RELT expression and pathological grading in pan-cancer; **(K)** K-M curves of high and low RELT expression within Stage IV grading of ccRCC affecting patients’ prognosis; **(L)** GO enrichment analysis of the DEGs; **(M)** KEGG enrichment analysis of the DEGs; **(N)** GSEA enrichment analysis of the DEGs; **(O)** PPI network graph of the top 10 proteins associated with RELT in the STRING online platform; **(P)** Scatter plot of related genes in the PPI network map; **(Q)** Scatter plots of RELT expression levels versus tumor purity in ccRCC; **(R)** Correlation analysis of RELT with immune cells; **(S)** Relationship between RELT expression and immune cells in pan-cancers under the CIBERSORT, EPIC, and TIMER algorithms; **(T)** Box plots of the expression of 66 immune cells in high and low expression subgroups of RELT under the xCELL algorithm; *P<0.05, **P<0.01, ***P<0.001.

Supplementary Figure 2 Scatterplot of RELT correlation with immune-related genes. **(A)** Scatterplot of RELT expression correlation with immune agonists; **(B)** Scatterplot of RELT expression correlation with immunosuppressants; **(C)** Scatterplot of RELT expression correlation with chemokines; **(D)** Scatterplot of RELT expression correlation with chemokine receptors; ***P<0.001.
