# Supplementary figures and images for: Immune-related RELT drives clear cell renal cell carcinoma progression through JAK/STAT signaling pathway activation
Source: Front Immunol. 2025 Nov 25;16:1659119. doi: 10.3389/fimmu.2025.1659119 (PMC12685935; doi:10.3389/fimmu.2025.1659119)

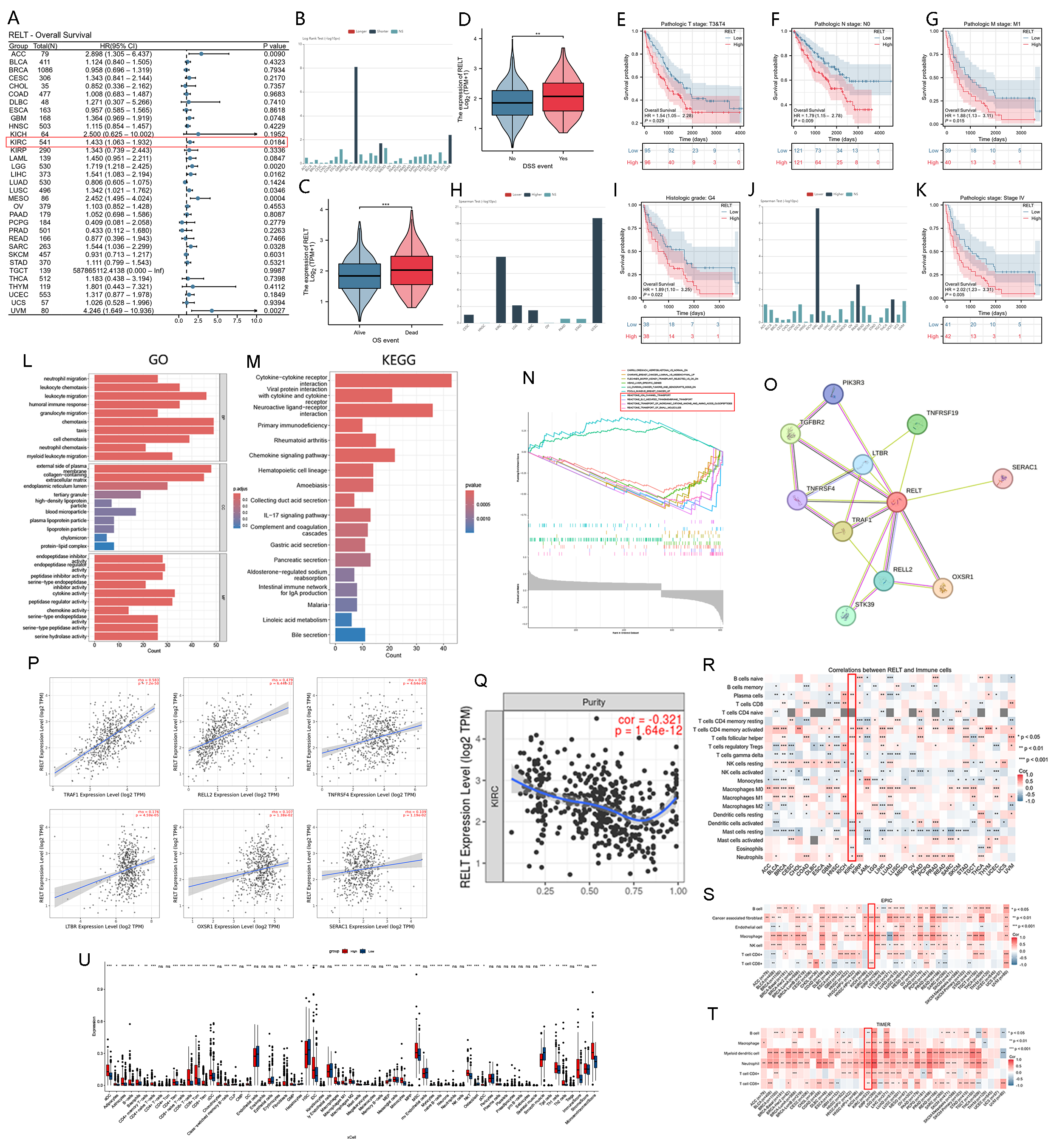

Supplement: Supplementary Figure 1 — Supplementary analysis on the role, relevance, and immune infiltration of RELT with respect to prognosis. (A) Forest plot of RELT overall survival in pan-cancer; (B, C) RELT expression was associated with clinical forest plots and grouped image heatmaps of pathological characteristics; (D) relationship between RELT expression and overall survival in pan-cancer; (E) box plot of RELT expression versus overall survival in ccRCC; (F) box plot of RELT expression versus DSS in ccRCC; (G) high and low RELT expression within the T3, T4, N0, and M1 stages of ccRCC affecting the prognosis of the patients’ K-M curves; (H) Relationship between RELT expression and histological grading in pan-cancer; (I) K-M curves of high and low RELT expression within G4 grading of ccRCC affecting patients’ prognosis; (J) Relationship between RELT expression and pathological grading in pan-cancer; (K) K-M curves of high and low RELT expression within Stage IV grading of ccRCC affecting patients’ prognosis; (L) GO enrichment analysis of the DEGs; (M) KEGG enrichment analysis of the DEGs; (N) GSEA enrichment analysis of the DEGs; (O) PPI network graph of the top 10 proteins associated with RELT in the STRING online platform; (P) Scatter plot of related genes in the PPI network map; (Q) Scatter plots of RELT expression levels versus tumor purity in ccRCC; (R) Correlation analysis of RELT with immune cells; (S) Relationship between RELT expression and immune cells in pan-cancers under the CIBERSORT, EPIC, and TIMER algorithms; (T) Box plots of the expression of 66 immune cells in high and low expression subgroups of RELT under the xCELL algorithm; *P<0.05, **P<0.01, ***P<0.001. [file Image1.tiff]

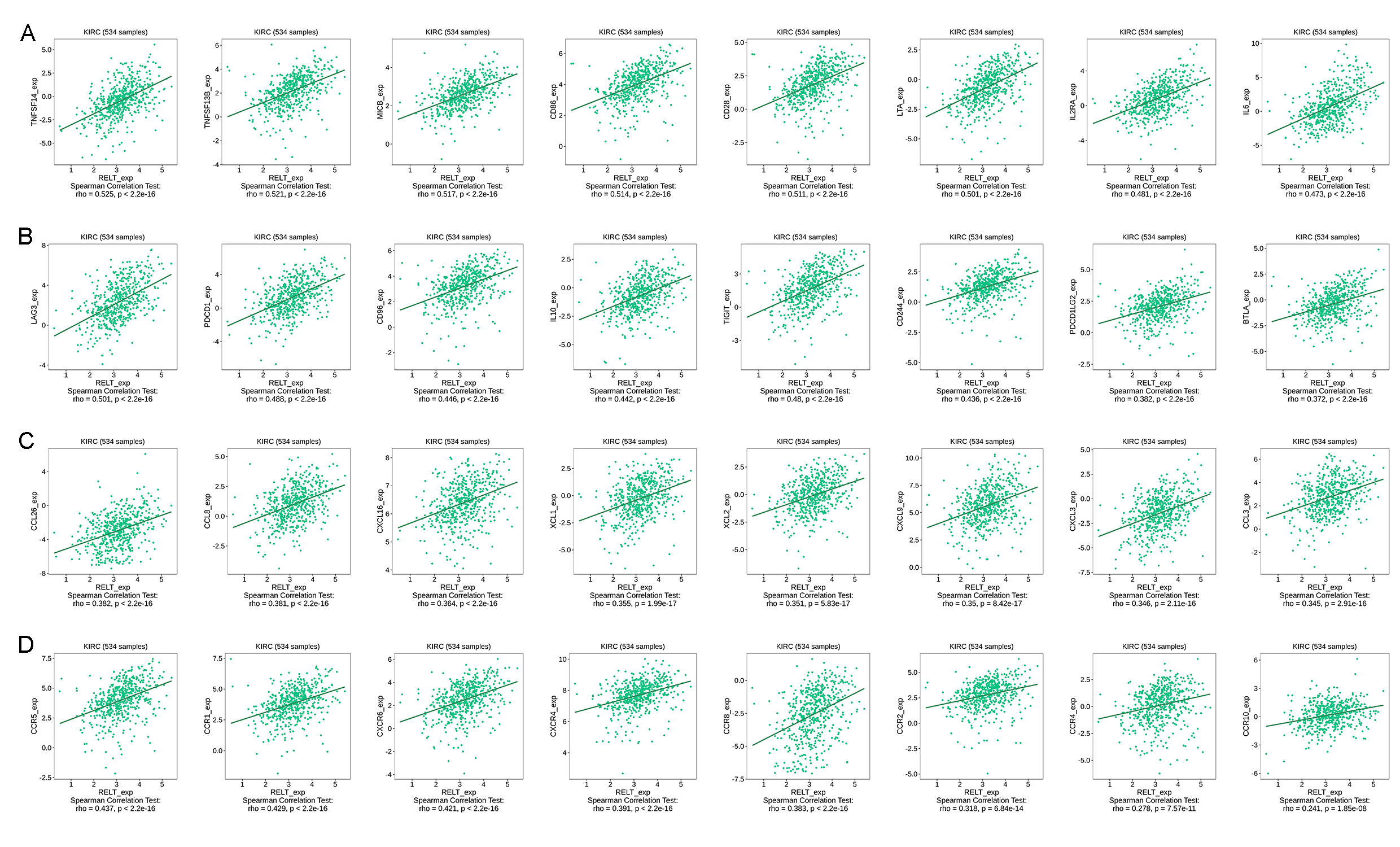

Supplement: Supplementary Figure 2 — Scatterplot of RELT correlation with immune-related genes. (A) Scatterplot of RELT expression correlation with immune agonists; (B) Scatterplot of RELT expression correlation with immunosuppressants; (C) Scatterplot of RELT expression correlation with chemokines; (D) Scatterplot of RELT expression correlation with chemokine receptors; ***P<0.001. [file Image2.tiff]
